# Automation in clinical chemistry: developments and recent trends

**DOI:** 10.1155/S1463924678000048

**Published:** 1978

**Authors:** F. L. Mitchell

**Affiliations:** Division of Clinical Chemistry Clinical Research Centre Watford Road Harrow, Middlesex HA1 3 UJ UK


### Automation in clinical chemistry: developments and recent trends

**F. L. Mitchell**

*Division of Clinical Chemistry, Clinical Research Centre, Watford Road, Harrow, Middlesex, HA1 3UJ, UK.*

**SINCE** the late 1940's the workload of many clinical chemistry laboratories has doubled every 4 years and this alone would have provided a stimulus to mechanise, but with the added advantage of the type of work involved forming a particularly fertile base for automation, it is not surprising that in the clinical application of chemistry, automation as we now know it for laboratory work was born, and most of the subsequent advances have been made.

There are several reasons why the automation of clinical chemistry is not difficult: the samples involved, mostly blood, but also urine and other body fluids, are liquid; the substances to be measured are largely present within clearly defined limits; the load of work is high and regular; world-wide the financial returns on work done can be high, and above all a rapid and reliable laboratory service is now a vital constituent of health care.

The impact which was first made by the continuous flow approach invented by Dr. Skeggs and introduced by Technicon in the 1950's has now become history. However, in the first issue of a journal with a readership covering many non-clinical areas of chemistry, it is pertinent to review for the benefit of all, some of the philosophies and developments which have revolutionised the clinical chemistry laboratory. For many industrial applications chemical automation has developed independently, for example, the use of gas chromatography in the automation of major functions in the petroleum industry, and other aspects generally have been covered by Dr. Stockwell in this Journal (1978).

### Principles used

While the continuous flow system was being developed and exploited by Technicon, other companies concentrated upon the development of the so-called discrete analyser, where samples are dealt with in separate tubes. In many ways, the discrete approach mimics the manipulations carried out in manual chemistry utilising pipettes, test tubes, etc. Developments in the two systems have moved in parallel, and important criteria such as accuracy, precision, required volumes of sample and reagent, etc., have improved roughly at the same rate in successive instruments utilising both principles of automation. Ingenuity has allowed both principles to be applied to difficult techniques such as radioimmunoassay, but though kinetic measurements are possible using continuous flow, there is no doubt that in this area discrete analysis systems offer far more scope. It is becoming realised that for many assays, kinetic measurements covering either short of long periods, offer considerable advantages over end-point measurements (Greinke and Mark, 1978), and it could be that an increasing need for kinetic monitoring will lead in the future to a general movement in favour of discrete analysers.

### Emergency work

Much work in the clinical laboratory is required for emergency situations occurring outside the normal working hours of the laboratory; most large automatic analysers operate most efficiently when carrying out long runs of assays and start-up for a single test is not usually practical. Emergency work is therefore often done manually when the operator is tired or harassed, and the maximum chance of human error is introduced at the very time when a life is most likely to be at stake. It is interesting that the two major instruments specially designed for this purpose employ the discrete principle. The Du Pont ACA (Automatic Clinical Analyser) has been available for many years, and in the recently introduced Technicon STAC (Single Test Analyser with Computer), the company has discarded the continuous flow principle for the first time in a major instrument. Both machines utilise individual chemistry packages for each measurement. A plastic bag in the case of the ACA and a rigid disposable container in the case of the STAC act as reagent container, reaction container and cuvette. This type of instrument can also be used for normal laboratory working, but the cost of individual packages means that overall cost effectiveness needs careful consideration.

Many companies now sell reagents to use with their machines, and increasingly the choice of chemistry for a particular assay has tended to move from the operator to the instrument manufacturer. In the case of the ACA and STAC, the control of the manufacturer over the chemistry used is absolute, but in many other machines there is the option of accepting the manufacturers' reagents or making them up independently. Being completely in the hands of the manufacturer understandably offends the professional feelings of many chemists, but advantages lie in the very careful quality control which a responsible manufacturer can build into his reagent manufacturing processes; the more work it is possible to do in this way, the more time the clinical chemist should be able to devote to more difficult work, further development and clinical interpretation problems.

### Rotary analysers

Requirements to carry out biochemical measurements in the absence of gravity in space, led to the development by Dr. Anderson at the Oakridge National Laboratory, Tennessee, of the GeMSAEC (General Medical Sciences — Atomic Energy Commission) type of centrifugal analyser (1969). In this, samples and reagents are added near the centre of a centrifuge rotor; on rotation all move to, and mix in cuvettes at the periphery where the chemical reaction proceeds and optical monitoring takes place as each cuvette cuts a light beam. Mixing in all channels of the rotor occurs simultaneously and very quickly, and with optical density readings being obtained during every revolution, the system is ideal for kinetic measurements. It suffers however, the disadvantage of being discontinuous in that the rotor has to be stopped for reloading; it is also difficult to carry out more than one type of assay in one rotor at any one time, although this can be overcome with a penalty on cost, by using disposable rotors containing prepackaged chemistry.

The problem of discontinuity with the GeMSAEC analyser has been overcome in the DACOS (Discrete Analyser with Continuous Optical Scanning) principle shortly to be available in an instrument under development by Coulter Electronics Inc. Here, the reaction tubes are situated at the periphery of a rotor which only rotates slowly in a stepping manner, to enable the tubes to be loaded with sample and subsequently washed when measurements are complete. Instead of the tubes rotating rapidly through the light beam, the light beam rotates on the same axis as the rotor, and the signal pattern from the detector is therefore identical to that from an instrument employing the GeMSAEC principle. Long reaction times can be accommodated either by slowing down the stepping motion of the rotor, or suppressing the washing and sample and reagent addition systems for one or more cycles. The scanning speed of the light beam can be varied within very wide limits. It would appear that this instrument combines the high sample throughput potential of the discrete analyser, with the kinetic measurement versatility of the GeMSAEC principle.

### Multichannel operation

Clinical requirements often demand the measurement of many substances on any one biological specimen. With the advent of multi-channel analysers employing both the continuous flow and discrete principles, came the ability to carry out not only specifically requested assays, but to perform them cheaply as part of the same profile of analyses on all specimens (discretionary and non-discretionary multichannel analysis). This facility led to the so-called 'screening' for the concentrations of many analytes not only for sick patients, but for healthy individuals. Much debate has taken place as to the value of this procedure; major problems being to decide what limits to apply for a particular measurement to be considered abnormal, and whether or not an abnormal measurement shall be acted upon medically. Over indulgence by the clinician in responding to such results can lead to considerable problems, yet ignoring any one finding might lead to retrospective litigation if a patient can prove that action at the time could have prevented a serious illness.

The current trend is to return to discretionary analysis, and relatively small, compact high-speed instruments for this purpose are becoming available. Their operation is practicable because of the development of easily worked computerised test selection mechanisms; full sample identification allows instrument programming to be carried out independently of the placing sequence of the samples, and instrument memory allows results to be requested at any time after the analyses have been completed.

Perhaps the most important effects automation has had in clinical chemistry, apart from increasing work throughput, have been the elimination of much human error in the form of making "blunders", i.e. mixing specimens, faulty transcription of results, etc., and improving the overall precision by replacing human variations in pipette operating, etc., with mechanically reproducibility.

### Layer chemistry

In the relatively snort time that chemistry has been applied to the biological field, and particularly to medicine, biochemists have introduced several new and dramatic approaches; layer chemistry follows after notably, continuous flow automation, gas chromatography and radioimmunoassay. It is remarkable how much of the chemistry done in clinical laboratories, can with certain modifications, be carried out easily and simply in dry-to-the-touch separate but interacting layers on a supporting medium (Curme *et al.*, 1978; Spayd *et al.*, 1978). Factory procedures have been perfected to reproduce the content of up to 16 layers on a photographic colour film with a high degree of precision; though photographic chemistry is not involved, the principles are transferable to the analysis of blood.

A small drop of serum is placed on the surface of a small piece of film which has been termed a chip. The make-up of the first (spreading) layer is such that the serum spot spreads evenly, and the constituents for assay pass into the next layer. The volume of serum is not therefore highly critical and one advantage of the technique is the removal of the need to measure the sample with a high degree of accuracy. Reagents have been placed during manufacture in the different layers of the film, so that the various stages of the assay can proceed as the analyte permeates downwards. Finally, a colour is developed in the last layer and measurement is carried out by reflectance densitometry.

Many advantages of this new approach are immediately evident, the chips are easily handled, and it will be possible to store a large number of different types in a multichannel instrument. The making up and storage of aqueous reagents, together with the necessity, when carrying out assays, accurately to measure large numbers of reagents, are all eliminated. Most of the accuracy and precision of measurement become the responsibility of the manufacturers.

Photographic film of high quality has been in production for many years and the high precision which can be obtained with clinical chemistry measurements using the layer techniques is undoubtedly due to an ability to draw on decades of manufacturing experience, firstly in black and white and then in colour photography.

If this approach is as successful as it would appear, it could be that in the not far distant future the use of diluents and tubes as at present understood in our laboratories, might be dispensed with for many purposes in clinical chemistry. Parallel with this however, since the technology is so specialised, will be an acceleration of the present trend towards the transfer of chemistry development expertise from the assay laboratory, to the manufacturer of diagnostic materials.

### Data processing and laboratory management

With the installation of increasingly efficient automation, the problems in the laboratory, like the modern laundry, changed from doing the actual analytical work (or the washing) to the handling of incoming specimens, quality control and the printing and despatch of results. Much of this aspect of the work has been taken over highly efficiently in most laboratories of any size by computer. It has been hoped in several countries and by several industrial companies, that a computer sytem or systems might be devised which have fairly universal applicability. However, the varying conditions and requirements in different laboratories have proved to be such that no one system has found universal acceptance without fairly major changes.

Difficulties commence with the degree of computerisation which the hospital itself, and the other departments of pathology, are prepared to accept. The ultimate system will undoubtedly be one where machine-readable patient identification commences with the first attendance of the patient at the hospital. The full identity plus information on any work done on the patient would be held in the hospital's computerised data-bank to which all the laboratories would have access. Machine-readable identity would accompany every specimen and laboratory functions would be managed by a relatively small "front end" processor, situated in the laboratory, but with full access to the main hospital computer. The laboratory system would be capable of interrogating the main hospital system to produce on visual display units in the laboratory, all previous work done on any particular patient.

The main advantage of laboratory computerisation has undoubtedly proved to be the efficient and speedy handling of large workloads and the efficient production of hundreds of reports within the very short time available between completion of the work and the deadline for despatch of reports to wards, etc.

By no means least of the advantages however, has been a dramatic reduction in the number of random errors. The number of specimen manipulations and data transfers involved in any particular assay can be considerable, and without the installation of automatic chemistry with computerised data handling it has been estimated that gross errors can occur in up to 5% of specimens handled. Statistics of this nature are difficult to obtain simply because the errors are indeed of random nature and origin, however, it is accepted that with the maximum amount of mechanisation and computerisation, they can be reduced overall to less than 1%. This figure might still be considered to be high by some, but it must be remembered that it is difficult for example, to prevent a doctor taking a specimen from the wrong patient, or putting the wrong machine-readable label on a tube.

Another major advantage has proved to be in the area of quality control. The computer can prepare special listings with quality control specimens identified, for the quality control officer to check before data are released for reporting. The computer can indeed be programmed to identify a quality control problem without human intervention, and notify those concerned. At the end of a defined period it can produce statistical data on the quality control performance of the laboratory.

A major task for senior laboratory personnel has always been the inspection and signing of reports at the end of the day. The object of this is to detect any gross errors which might be apparent, give any clinically relevant comment, and if necessary, draw attention to any clinical action required. The computer can be programmed very satisfactorily to deal with much of this work, and "sign" a large proportion of the forms, drawing attention only to those where human intervention is required. Dealing with the small number of forms thus extracted is even then greatly facilitated by the ability to call up on display all previous work done on a particular patient. If this facility is not available on a ward, the same advantage can be transferred to the clinician by "cumulative reporting", where the computer prints out for any particular patient, not only the report of the day, but also all the relevant work done previously.

It might be concluded that mechanisation and computerisation can solve many laboratory problems involved with the well tried and established techniques in use; but it must be remembered that computers and machines are not chemists and the important problem of chemical accuracy still lies firmly in human control. Mechanical and electronic systems can be made to control and report on precision in highly sophisticated ways, but the question as to whether the figures reported reflect the true content of the sample rests with the chemistry involved. That major problems in this area still exist is shown by the alarming differences in results for some analytes which can be obtained on the same specimen in different laboratories. When the underlying problems have been solved, mechanics and electronics can undoubtedly help with control, but years of work lie ahead before the responsible clinical chemist can be happy with his accuracy control.

### Instrument simplification

The instrumental advances so far considered have taken place almost entirely in and for countries with highly developed technologies and economies. The trend has been towards ever more complex, faster, and inevitably more expensive machines. Undoubtedly a competitive element creeps in, in that it can so easily be considered out of fashion not to have a highly complex computerised laboratory system. There is no doubt that in proceeding as we have done over recent years, it is all too easy to forget that bigger is not always better and that electronics can be used to simplify as well as make more complex. The development of the electronic calculator is an excellent example where complex calculations can now be carried out with albeit, extremely complex circuitry, but packaged in such a way as to be robust, simply operated and above all, cheap, and capable of running for long periods from an integral battery. The same principles can be applied to the operation of the chemistry colorimeter. The light source can be a flashing light-emitting diode, and the read-out digital instead of analogue. The circuitry can be arranged so that read-out for a variety of analytes can be in concentration units. The whole can be made to operate off a small integral battery which would last almost as long as in an electronic calculator. Encapsulation of most of the working parts would largely ensure independence from environmental conditions, and there is no reason why such an instrument could not be carried in the pocket and utilised with simple packaged chemistry, not only in developing countries where independence from electrical supply, servicing, etc., is important, but also for the many occasions world-wide where analyses are required, but a central laboratory is not readily accessible.

If the definition of automation is "when some manual involvement is removed by the use of a mechanism, be it computer or otherwise" (Stockwell, 1978), then this approach may be classified as automation just as surely as the development of complex computer controlled multichannel analysers.

REFERENCES

Anderson, N. G. (1969) *Analytical Biochemistry*, **28**, 545-562.

Curme, H. G., Columbus, R. L., Dappen, G. M., Eder, T. W., Fellows, W. D., Figueras, J., Glover, C. P., Goffe, C. A., Hill, D. E., Lawton, W. H., Muka, E. J., Pinney, J. E., Rand, R. N., Sanford, K. J. and Wu, T. W., (1978) *Clinical Chemistry*, **24**, 1335-1342.

Greinke, R. A. and Mark, H. B. (1978) *Analytical Chemistry*, **50**, 70R-76R.

Spayd, R. W., Bruschi, B., Burdick, B. A., Dappen, G. M., Eikenberry, J. N., Esders, T. W., Figueras, J., Goodhue, C. T., LaRossa, D. D., Nelson, R. W., Rand, R. N. and Wu, T. W. (1978) *Clinical Chemistry*, **24**, 1343-1350.

Stockwell, P. B. (1978) *The Journal of Automatic Chemistry*, **1**, 10.

The following papers are expected to be published in forthcoming issues of *The Journal of Automatic Chemistry.*

A computer controlled multiplexed absorptiometer for reaction rate analysis *by M. Snook* et al.

Continuous monitoring using polarographic electrodes *by L. C. Clark.*

Automatic system for quality control of dyestuffs: design philosophy and implementation *by H. C. Metz and G. Michel.*

Teaching a graduate course in automatic chemistry *by R. F. Browner.*

Specification for prototype automatic instrumentation *by J. E. Carlyle.*

Design considerations involved in the development of an automatic hydride evolution system for the measurement of nanogram amounts of arsnic, selenium and antimony by atomic spectroscopy *by D. G. Porter and A. L. Dennis.*

The use if infrared reflectance in dietry measurements of animal feeds *by H. Swann.*

